# Prevalence, Cost, and Variation in Cost of Pediatric Hospitalizations in Ontario, Canada

**DOI:** 10.1001/jamanetworkopen.2021.47447

**Published:** 2022-02-09

**Authors:** Peter J. Gill, Thaksha Thavam, Mohammed Rashidul Anwar, Jingqin Zhu, Patricia C. Parkin, Eyal Cohen, Teresa To, Sanjay Mahant, Francine Buchanan, Wenjia Chen, Ronald Cohn, Mairead Green, Matt Hall, Kate Langrish, Colin Macarthur, Myla Moretti, Michelle Quinlan, Ann Bayliss, Ronik Kanani, Sean Murray, Catherine Pound, Mahmoud Sakran, Anupam Sehgal, Sepi Taheri, Gita Wahi

**Affiliations:** 1 Department of Pediatrics, University of Toronto, Toronto, Ontario, Canada; 2 The Hospital for Sick Children, Toronto, Ontario, Canada; 3 Child Health Evaluative Sciences, SickKids Research Institute, Toronto, Ontario, Canada; 4 Institute of Health Policy, Management and Evaluation, Dalla Lana School of Public Health, The University of Toronto, Toronto, Ontario, Canada; 5 Saw Swee Hock School of Public Health, National University of Singapore, Singapore; 6 Children’s Hospital of Eastern Ontario, Ottawa, Ontario, Canada; 7 Children's Hospital Association, Lenexa, Kansas; 8 Children’s Health Division, Trillium Health Partners, Mississauga, Ontario, Canada; 9 Department of Pediatrics, North York General Hospital, Toronto, Ontario, Canada; 10 Department of Pediatrics, Northern Ontario School of Medicine, Sudbury, Ontario, Canada; 11 University of Ottawa, Ottawa, Ontario, Canada; 12 Department of Pediatrics, Queens University, Kingston, Ontario, Canada; 13 Department of Pediatrics, Lakeridge Health, Oshawa, Ontario, Canada; 14 Department of Pediatrics, Western University, London, Ontario, Canada; 15 Division of General Pediatrics, Department of Pediatrics, McMaster University and McMaster Children’s Hospital, Hamilton, Ontario, Canada

## Abstract

**Importance:**

Identifying conditions that could be prioritized for research based on health care system burden is important for developing a research agenda for the care of hospitalized children. However, existing prioritization studies are decades old or do not include data from both pediatric and general hospitals.

**Objective:**

To assess the prevalence, cost, and variation in cost of pediatric hospitalizations at all general and pediatric hospitals in Ontario, Canada, with the aim of identifying conditions that could be prioritized for future research.

**Design, Setting, and Participants:**

This population-based cross-sectional study used health administrative data from 165 general and pediatric hospitals in Ontario, Canada. Children younger than 18 years with an inpatient hospital encounter between April 1, 2014, and March 31, 2019, were included.

**Main Outcomes and Measures:**

Condition-specific prevalence, cost of pediatric hospitalizations, and condition-specific variation in cost per inpatient encounter across hospitals. Variation in cost was evaluated using (1) intraclass correlation coefficient (ICC) and (2) number of outlier hospitals. Costs were adjusted for inflation to 2018 US dollars.

**Results:**

Overall, 627 314 inpatient hospital encounters (44.8% among children younger than 30 days and 53.0% among boys) at 165 hospitals (157 general and 8 pediatric) costing $3.3 billion were identified. A total of 408 003 hospitalizations (65.0%) and $1.4 billion (43.8%) in total costs occurred at general hospitals. Among the 50 most prevalent and 50 most costly conditions (of 68 total conditions), the top 10 highest-cost conditions accounted for 55.5% of all costs and 48.6% of all encounters. The conditions with highest prevalence and cost included low birth weight (86.2 per 1000 encounters; $676.3 million), preterm newborn (38.0 per 1000 encounters; $137.4 million), major depressive disorder (20.7 per 1000 encounters; $78.3 million), pneumonia (27.3 per 1000 encounters; $71.6 million), other perinatal conditions (68.0 per 1000 encounters; $65.8 million), bronchiolitis (25.4 per 1000 encounters; $54.6 million), and neonatal hyperbilirubinemia (47.9 per 1000 encounters; $46.7 million). The highest variation in cost per encounter among the most costly medical conditions was observed for 2 mental health conditions (other mental health disorders [ICC, 0.28] and anxiety disorders [ICC, 0.19]) and 3 newborn conditions (intrauterine hypoxia and birth asphyxia [ICC, 0.27], other perinatal conditions [ICC, 0.17], and surfactant deficiency disorder [ICC, 0.17]).

**Conclusions and Relevance:**

This population-based cross-sectional study of hospitalized children identified several newborn and mental health conditions as having the highest prevalence, cost, and variation in cost across hospitals. Findings of this study can be used to develop a research agenda for the care of hospitalized children that includes general hospitals and to ultimately build a more substantial evidence base and improve patient outcomes.

## Key Points

**Question:**

What conditions should be prioritized for research involving hospitalized children?

**Findings:**

In this cross-sectional study of 627 314 inpatient encounters among children at 165 hospitals in Ontario, Canada, 65.0% of encounters and 43.8% of costs occurred at general hospitals. Several newborn and mental health conditions were identified as most prevalent, costly, and with high variation in cost across hospitals.

**Meaning:**

The results of this study can be used to design a research agenda for the care of hospitalized children that includes general hospitals and may inform researchers, health care system leaders, and funders about research areas and quality improvement efforts that could be prioritized.

## Teaser

This cross-sectional study assesses the prevalence, cost, and variation in cost of pediatric hospitalizations at all general and pediatric hospitals in Ontario, Canada, and identifies conditions that may be prioritized for future research.

## Introduction

Hospital care costs account for the largest component of all health care costs.^1,2^ Among children, hospital expenditures are high, with costs increasing over time.^3,4^ Despite the large volume and high cost of admissions, hospitalized children may not receive optimal care owing to a lack of high-quality evidence.^5,6,7^ For example, few randomized clinical trials have been conducted in pediatric hospitals compared with specialty units, such as oncology^8^ and critical care.^9,10^ The lack of high-quality research can produce unnecessary variation in clinical practice and outcomes.^11,12,13,14^

Identifying topics to prioritize for future research is necessary to build a meaningful research agenda for the care of hospitalized children. One factor identified in research prioritization frameworks is the condition-related criterion, which includes assessing the prevalence, cost, and variation in cost of the illness.^15,16^ Conditions that are common, require costly care, and have high cost variation increase in rank to become high-priority conditions for research.^15,16^ Existing studies have examined the burden of pediatric hospitalizations in North America,^16,17,18,19,20^ but study limitations have made it difficult to use their findings for future research. In the US, 3 studies^16,17,18^ have identified common and costly conditions among hospitalized children. The most recent study^18^ excluded general hospitals, in which most pediatric hospitalizations occur.^17^ In Canada, 2 studies^19,20^ identified the most common reasons for pediatric hospitalization; however, those studies used data that are now more than 2 decades old and did not explore the cost of conditions.

Therefore, the aim of this study was to describe the epidemiologic characteristics of pediatric hospitalizations and identify conditions that could be prioritized for research using population-based data across all general and pediatric hospitals in Ontario, Canada. The specific objectives were to (1) assess the condition-specific prevalence, cumulative cost, and variation in cost of pediatric hospitalizations and (2) rank order the pediatric hospital conditions to identify those with high prevalence, cost, and/or variation in cost as priorities for future research involving hospitalized children.

## Methods

### Study Design

This population-based cross-sectional study focused on children with hospital encounters in Ontario, the most populous province in Canada with a population of 14.8 million.^21^ Data were obtained from linked Ontario health administrative databases housed at ICES (formerly known as the Institute for Clinical Evaluative Sciences). The data included all children eligible for provincial health care coverage and all general and pediatric hospitals in Ontario. The study was approved by the Hospital for Sick Children Research Ethics Board with a waiver of informed consent because deidentified data were used. This study followed the Strengthening the Reporting of Observational Studies in Epidemiology (STROBE) reporting guideline for cross-sectional studies.^22^

### Data Sources

Clinical information and intensity of resources consumed for hospital encounters (inpatient discharges or same-day surgery) were obtained from the Canadian Institute for Health Information Discharge Abstract Database and the Same Day Surgery Database. Demographic information was extracted from the Registered Persons Database,^23^ and annual population estimates for Ontario were obtained from intercensal population estimates and projections for local health integrated networks.^24^

### Study Population

Children younger than 18 years with an inpatient hospital encounter between April 1, 2014, and March 31, 2019, were included. We excluded encounters among children with missing or invalid birth dates, death dates, or discharge dates; encounters among non-Ontario residents; encounters indicating normal newborn birth based on a most responsible diagnostic code (defined as the condition that contributes the greatest extent to the patient's hospital stay) from the *International Statistical Classification of Diseases and Related Health Problems, Tenth Revision, Canada* (*ICD-10-CA*); and same-day surgery encounters (all exclusions are shown in the eFigure in the Supplement).

### Pediatric Clinical Classification System

For each hospital encounter, the most responsible diagnosis was determined using the *ICD-10-CA* discharge diagnostic code and classified into discrete clinical condition categories using codes from the Pediatric Clinical Classification System. This system classifies 16 992 *ICD-10-CA* codes into 781 clinically meaningful categories to identify specific pediatric conditions, including treatments (eg, chemotherapy). The Pediatric Clinical Classification System was first developed for the US *International Statistical Classification of Diseases, Tenth Revision, Clinical Modification (ICD-10-CM)* coding system and evaluated using US administrative pediatric hospitalization data.^25^ This grouping system categorized conditions into medical, surgical, and medical/surgical based on the percentage of encounters for the condition that had a surgical procedure code. If fewer than 30% of encounters for the condition had a surgical procedure code, the condition was classified as medical; if more than 70% of encounters for the condition had a surgical procedure code, the condition was classified as surgical; and if between 30% and 70% of encounters for the condition had a surgical procedure code, the condition was classified as medical/surgical.^18^

### Outcome Measures

We estimated the condition-specific prevalence of hospitalization for each condition, with condition-specific prevalence defined as the proportion of hospital encounters owing to the specific condition over the study period. We also calculated the condition-specific cost of hospitalization for each condition as a proxy for the volume of resources used to manage the condition.^16^ Costs were estimated using an ICES costing algorithm, which multiplied the resource intensity weight assigned to each inpatient case recorded in the Canadian Institute for Health Information Discharge Abstract Database with the provincial mean unit cost of acute hospitalization (ie, cost per weighted case) for the corresponding fiscal year.^26^ The resource intensity weight represents the amount of resources used by the inpatient case relative to an average inpatient case.^26^ Costs were adjusted for inflation to 2018 US dollars (mean exchange rate: $0.77 US dollars = $1.00 Canadian dollar). Details on the case-costing methods are available online.^26^ We also estimated the condition-specific variation in cost per encounter across hospitals to measure the variation in resources used to manage each condition across hospitals.^16^

### Patient, Encounter, and Hospital Characteristics

Patient characteristics included age at the time of the hospital encounter (<30 days, ≥30 days to <1 year, 1-4 years, 5-12 years, and 13 to <18 years),^16^ sex, and rural vs urban residence (measured using the Rurality Index for Ontario, with urban defined as a score <40 and rural defined as a score ≥40).^27,28^ The components used to calculate the Rurality Index for Ontario included community population size and density as well as travel time to the nearest basic and advanced referral centers.^27^ The number of complex chronic conditions (CCCs) present (0, 1, 2, or ≥3)^29,30^ for each patient was estimated using the diagnostic and procedural codes adopted by the Canadian Institute for Health Information.^31^ To identify whether CCCs were present, for each encounter, the patient’s hospital data were examined for the previous 2 years or until birth if the patient was younger than 2 years. To measure the socioeconomic status of the cohort, the Ontario Marginalization Index was used to identify material deprivation^32^ by quintile (with quintile 1 indicating least deprived and quintile 5 indicating most deprived). The factors used to measure material deprivation were income, quality of housing, educational level, and family structure characteristics.^32,33^ For hospital encounter characteristics, we identified the condition type (medical, surgical, or medical/surgical) and length of stay in days.

For hospital characteristics, we identified hospital type, which was categorized as general or pediatric (defined as a tertiary care children’s hospital or a hospital with a level 3 neonatal intensive care unit). The hospital region (central east, central south, central west, east, north, southwest, or Toronto, Ontario) and the mean volume of inpatient encounters per year were also identified.

### Statistical Analysis

For each hospital condition, we determined the condition-specific prevalence over the study period using the number of hospital encounters associated with the condition as the numerator and the total number of encounters associated with all conditions as the denominator. We calculated the prevalence per 1000 patient encounters. The median and mean cost per encounter and the cumulative cost of encounters for each condition were also calculated. Hospital conditions were ranked from highest to lowest based on the total volume of encounters (called prevalence rank) and based on cumulative cost.

For the 25 medical conditions with the highest cumulative cost, we determined the variation in cost per encounter across hospitals. We focused on the conditions with the highest cumulative cost because they would either be highly prevalent or highly costly per encounter.^16^ The condition-specific variation in cost across hospitals was adjusted for known factors associated with variation in cost, including age, sex, number of CCCs present, material deprivation, and rural vs urban classification to minimize confounding from other factors that may have biased the extent of variation in cost across hospitals.^16,34,35,36^

To alleviate skewness, hospital encounter costs were log transformed before conducting the analysis of variation in cost. The variation in cost per encounter was assessed using 2 methods^16^: intraclass correlation coefficient (ICC) and number of outlier hospitals. To assess variation in cost per encounter using the ICC method, for each condition, the variation in cost (using cost per encounter) across hospitals was divided by the total variation in cost per encounter (which included within-hospital and across-hospital variation in costs). The ICC was calculated using a mixed-effects model with random intercepts for each hospital and patient characteristics as fixed effects.^16^ To assess variation in cost per encounter using the number of outlier hospitals, for each condition, the number of hospitals with more than 30% of encounters in the highest or lowest quintile of overall cost per encounter was calculated. The ICC was a single measure that quantified the extent of variation in cost across hospitals, whereas the number of outlier hospitals provided granularity regarding the number of hospitals that met the low-cost and/or high-cost outlier hospital criteria for the condition.^16^ To ensure we had an adequate sample of hospitals and encounters per hospital for the analysis of variation in cost, we restricted this analysis to conditions (from the 25 highest-cost medical conditions) that had data available from at least 10 hospitals with more than 25 encounters per hospital for the corresponding condition.^37^ Secondary analysis examined the variation in cost per encounter for each condition across general hospitals alone. This analysis was not performed separately across pediatric hospitals because of the small number of pediatric hospitals.

Additional analyses identified the 25 most prevalent and 25 most costly conditions in pediatric vs general hospitals. These analyses were conducted as differences in the volume, cost, and characteristics of pediatric hospitalizations reported between hospital types.^17^ Analyses were performed using SAS Enterprise Guide software, version 7.1 (SAS Institute Inc).

## Results

In total, there were 1 361 712 hospital encounters among children in Ontario between April 1, 2014, and March 31, 2019. After exclusions (437 166 of which were normal newborn births), 627 314 inpatient encounters among children were included from 157 general hospitals and 8 pediatric hospitals (eFigure in the Supplement). The total hospital cost was $3.3 billion, with 408 003 hospitalizations (65.0%) and $1.4 billion (43.8%) in total costs occurring at general hospitals.

Of the included encounters, 281 230 (44.8%) were among children younger than 30 days, 332 494 (53.0%) were among boys, and 571 437 (91.1%) were among children residing in urban areas (Table 1). Children with at least 1 CCC represented 141 653 inpatient encounters (22.6%). A total of 471 440 encounters (75.2%) occurred because of medical conditions, 94 727 encounters (15.1%) occurred because of surgical conditions, and 61 147 encounters (9.7%) occurred because of medical/surgical conditions. The median length of stay was 2 days (IQR, 1-4 days). The median annual hospital volume of inpatient encounters was 73.0 (IQR, 4.6-1003.2).

**Table 1.** Patient, Encounter, and Hospital Characteristics Among Children With Inpatient Encounters in Ontario, Canada, From 2014 to 2019

Characteristic	No. (%)
**Patients**	
Total inpatient encounters, No.	627 314
Age at time of encounter, median (IQR), y	0 (0-8.0)
Age range at time of encounter	
<30 d	281 230 (44.8)
≥30 d to <1 y	53 827 (8.6)
1 to 4 y	93 217 (14.9)
5 to 12 y	92 730 (14.8)
13 to <18 y	106 310 (16.9)
Sex	
Female	294 820 (47.0)
Male	332 494 (53.0)
Rurality of residence	
Rural	41 548 (6.6)
Urban	571 437 (91.1)
Missing	14 329 (2.3)
No. of complex chronic conditions present	
0	485 661 (77.4)
1	74 685 (11.9)
2	26 537 (4.2)
≥3	40 431 (6.4)
Material deprivation, quintile	
1 (Least deprived)	123 336 (19.7)
2	119 456 (19.0)
3	113 473 (18.1)
4	115 571 (18.4)
5 (Most deprived)	142 832 (22.8)
Missing	12 646 (2.0)
**Encounters**	
Type of condition	
Medical	471 440 (75.2)
Medical/surgical	61 147 (9.7)
Surgical	94 727 (15.1)
Length of stay, median (IQR), d	2.0 (1.0-4.0)
**Hospitals**	
Total hospitals, No.	165
Type of hospital	
Pediatric	8 (4.8)
General	157 (95.2)
Region	
Southwest	35 (21.2)
Central south	12 (7.3)
Central west	15 (9.1)
Central east	19 (11.5)
Toronto	14 (8.5)
East	27 (16.4)
North	43 (26.1)
Annual volume of inpatient encounters, median (IQR)	73.0 (4.6-1003.2)

### Prevalence and Cost

The 50 most prevalent and 50 most costly conditions (of 68 total conditions) ranked by cumulative cost are shown in Table 2. The top 10 most costly conditions accounted for 55.5% of all costs and 48.6% of all encounters among the 68 conditions. Extreme immaturity of newborn had the highest median cost per encounter ($21 702; IQR, $3698-$40 779). Low birth weight had the highest prevalence (86.2 per 1000 encounters). Several mental health conditions had the highest prevalence and highest cost among children; these conditions included major depressive disorder (20.7 per 1000 encounters; $78.3 million), adjustment disorders (12.1 per 1000 encounters; $40.9 million), anorexia nervosa (2.8 per 1000 encounters; $31.5 million), anxiety disorders (8.0 per 1000 encounters; $30.7 million), other mental health disorders (5.1 per 1000 encounters; $19.3 million), schizophrenia and psychotic disorders (2.4 per 1000 encounters; $17.2 million), and attention-deficit/hyperactivity disorder (3.5 per 1000 encounters; $16.3 million). Diagnoses within the condition category other mental health disorders are shown in eTable 1 in the Supplement.

**Table 2.** Prevalence and Cost for the 50 Most Costly and 50 Most Prevalent Conditions Among Children With Inpatient Encounters in Ontario, Canada, From 2014 to 2019

Condition^a^	Type of condition	Rank		Inpatient encounters, No.		Cost, $^b^	
		Based on total cost	Based on No. of encounters^c^	Total^d^	Prevalence^e^	Total	Per encounter, median (IQR)
Low birth weight	Medical	1	1	54 100	86.2	676 292 381	2924 (1236-13 777)
Preterm newborn	Medical	2	4	23 821	38.0	137 377 386	2468 (1255-6011)
Major depressive disorder	Medical	3	9	12 975	20.7	78 303 976	5780 (5306-6735)
Pneumonia	Medical	4	5	17 143	27.3	71 566 538	2929 (2709-4029)
Other perinatal conditions	Medical	5	2	42 674	68.0	65 791 674	1036 (1026-1236)
Bronchiolitis	Medical	6	6	15 950	25.4	54 581 261	2602 (2392-2700)
Surfactant deficiency disorder	Medical	7	43	3015	4.8	50 023 527	6645 (2148-15 952)
Neonatal hyperbilirubinemia	Medical	8	3	30 048	47.9	46 670 627	1447 (1064-1500)
Adjustment disorders	Medical	9	17	7621	12.1	40 888 157	5114 (4675-6314)
Transient tachypnea of newborn	Medical	10	7	14 287	22.8	40 298 745	1653 (1428-3383)
Chemotherapy	Medical	11	14	7905	12.6	37 529 210	4238 (2634-4490)
Drug withdrawal syndrome in newborn	Medical	12	39	3291	5.2	37 016 433	9473 (8713-13 274)
Anorexia nervosa	Medical	13	66	1758	2.8	31 464 629	18 398 (16 329-18 752)
Anxiety disorders	Medical	14	26	5010	8.0	30 680 583	6448 (5395-7030)
Asthma	Medical	15	8	13 449	21.4	28 454 191	1723 (1623-2081)
Acute appendicitis without peritonitis	Surgical	16	12	9978	15.9	27 902 739	2655 (2519-2832)
Feeding difficulties	Medical/surgical	17	22	6560	10.5	25 848 677	1531 (1036-4105)
Urinary tract infections	Medical	18	18	7088	11.3	25 645 046	3112 (2831-3466)
Other congenital anomalies	Surgical	19	42	3077	4.9	25 600 029	2474 (930-4716)
Infectious gastroenteritis	Medical	20	10	11 373	18.1	25 591 365	1735 (1706-1897)
Respiratory distress of newborn	Medical	21	16	7738	12.3	23 641 152	1541 (1428-2796)
Acute lymphoid leukemia	Medical	22	119	894	1.4	23 453 440	17 271 (12 208-29 469)
Necrotizing enterocolitis	Medical/surgical	23	174	459	0.7	23 045 352	17 821 (9370-47 819)
Acute appendicitis with peritonitis	Surgical	24	29	4400	7.0	22 170 824	4006 (3852-4755)
Complications of surgical procedures or medical care	Medical/surgical	25	30	4164	6.6	21 916 650	2393 (1438-4175)
Extreme immaturity of newborn	Medical	26	147	580	0.9	20 925 776	21 702 (3698-40 779)
Scoliosis	Surgical	27	101	1053	1.7	20 547 895	18 884 (12 943-19 834)
Intrauterine hypoxia and birth asphyxia	Medical	28	57	2326	3.7	20 469 981	3385 (1528-10 694)
Other mental health disorders	Medical	29	41	3168	5.1	19 272 426	2843 (2320-6185)
Sepsis of newborn	Medical	30	52	2638	4.2	18 806 085	4985 (2961-6845)
Neutropenia	Medical	31	44	3010	4.8	18 704 373	4240 (3703-6717)
Septicemia	Medical	32	61	1890	3.0	18 688 859	4630 (4117-8729)
Fracture of lower limb	Surgical	33	35	3776	6.0	18 566 246	3953 (3328-4926)
Respiratory failure	Medical	34	138	677	1.1	18 550 691	11 766 (4800-36 001)
Newborn respiratory failure	Medical	35	20	6642	10.6	18 328 994	1268 (1123-2335)
Neonatal hypoglycemia	Medical	36	15	7844	12.5	18 226 564	1078 (1030-2400)
Tetralogy of Fallot	Medical/surgical	37	147	580	0.9	17 923 567	17 303 (4047-28 984)
Schizophrenia and psychotic disorders	Medical	38	72	1519	2.4	17 244 815	10 768 (9374-11 384)
Transposition of great vessels	Medical/surgical	39	173	461	0.7	16 416 666	20 067 (3217-44 862)
Attention-deficit/hyperactivity disorder	Medical	40	58	2199	3.5	16 337 289	7046 (6367-7823)
Viral infection	Medical	41	21	6632	10.6	16 158 659	1918 (1829-2131)
Hypertrophy of tonsils and adenoids	Surgical	42	13	8166	13.0	15 948 718	1842 (1828-1907)
Intracranial injury	Medical	43	90	1156	1.8	15 775 600	3945 (2673-11 482)
Gastroschisis and exomphalos	Surgical	44	219	319	0.5	15 639 964	21 307 (1113-65 445)
Congenital tracheoesophageal disorders	Surgical	45	236	277	0.4	15 518 709	10 313 (2035-47 866)
Sleep apnea	Surgical	46	23	6358	10.1	15 498 436	1842 (1828-1907)
Hypoplastic left heart syndrome	Medical/surgical	47	253	238	0.4	15 261 396	14 977 (1595-54 180)
Screening for suspected conditions	Medical	48	11	10 817	17.2	14 444 254	1065 (1030-1236)
Cystic fibrosis	Medical	49	120	856	1.4	14 429 112	14 179 (12 436-16 173)
Seizures with and without intractable epilepsy	Medical	50	33	3913	6.2	14 356 766	1903 (1801-2754)
Cellulitis	Medical	51	31	4114	6.6	14 298 664	3055 (2482-3596)
Acute upper respiratory infection	Medical	52	25	5023	8.0	14 272 166	2057 (1967-2181)
Other lower respiratory disease	Medical	53	38	3533	5.6	14 126 940	2213 (1939-2791)
Fever of unknown origin	Medical	54	28	4693	7.5	12 746 513	2284 (2167-2529)
Infant of diabetic mother	Medical	55	19	6707	10.7	12 569 146	1078 (1030-1236)
Other aftercare	Medical/surgical	57	27	4738	7.6	11 353 620	1275 (1187-1554)
Fracture of upper limb	Surgical	61	32	3953	6.3	11 037 979	2931 (1984-3034)
Supracondylar fracture of humerus	Surgical	63	36	3713	5.9	10 643 826	2943 (2856-2974)
ABO hemolytic disease	Medical	81	34	3814	6.1	8 375 305	1858 (1682-2190)
Influenza	Medical	86	50	2865	4.6	8 212 883	2072 (1970-2393)
Diabetic ketoacidosis	Medical	87	46	2988	4.8	8 126 963	2607 (2272-2838)
Congenital genitourinary anomalies	Surgical	90	40	3171	5.1	7 717 287	930 (891-1629)
Neonatal tachycardia	Medical	93	48	2929	4.7	7 258 219	1078 (1030-1558)
Other convulsions	Medical	103	47	2954	4.7	6 568 239	1882 (1796-2198)
Respiratory problems after birth, other	Medical	110	49	2882	4.6	5 901 470	1526 (1428-1677)
Tongue-tied	Surgical	112	24	5128	8.2	5 892 461	1036 (1026-1078)
Birth trauma	Medical/surgical	118	45	3002	4.8	5 344 774	1065 (1026-1236)
Croup	Medical	124	37	3612	5.8	5 067 750	1148 (1103-1292)

Abbreviation: ABO, ABO blood group system.

^a^ Sixty-eight total conditions.

^b^ Costs adjusted for inflation to 2018 US dollars (mean 2018 exchange rate: $0.77 US dollars = $1.00 Canadian dollar).

^c^ Also known as prevalence rank in this study.

^d^ Total number of encounters for the condition over the study period.

^e^ Condition-specific prevalence per 1000 encounters.

**Figure 1.**
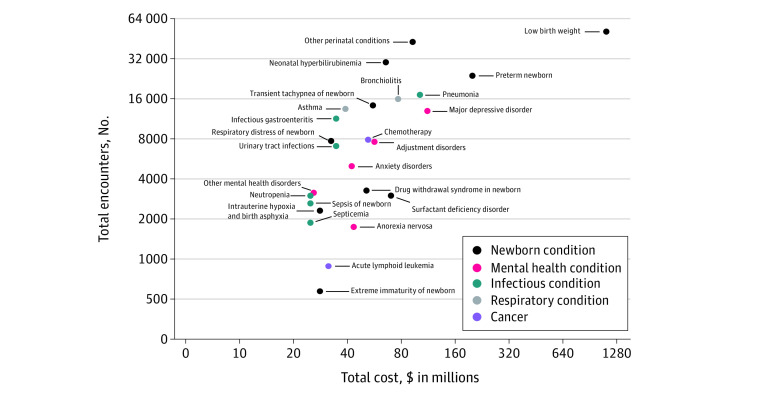
Volume and Cost of Encounters for the 25 Highest-Cost Medical Conditions Among Children With Inpatient Encounters in Ontario, Canada, From 2014 to 2019 Total costs (in millions) are presented in Canadian dollars. To convert Canadian dollars to US dollars (adjusted for inflation to 2018 US dollars), multiply values by 0.77. The x- and y-axes are presented using a log_2_ scale.

Of the 68 conditions, 7 were highly prevalent and costly: low birth weight (86.2 per 1000 encounters; $676.3 million), preterm newborn (38.0 per 1000 encounters; $137.4 million), major depressive disorder (20.7 per 1000 encounters; $78.3 million), pneumonia (27.3 per 1000 encounters; $71.6 million), other perinatal conditions (68.0 per 1000 encounters; $65.8 million), bronchiolitis (25.4 per 1000 encounters; $54.6 million), and neonatal hyperbilirubinemia (47.9 per 1000 encounters; $46.7 million) (Table 2; data for other perinatal conditions are shown in eTable 1 in the Supplement). The 25 most costly medical conditions were categorized into cancer (2 conditions: acute lymphoid leukemia and chemotherapy), newborn (10 conditions: drug withdrawal syndrome in newborn, extreme immaturity of newborn, intrauterine hypoxia and birth asphyxia, low birth weight, neonatal hyperbilirubinemia, other perinatal conditions, preterm newborn, respiratory distress of newborn, surfactant deficiency disorder, and transient tachypnea of newborn), mental health (5 conditions: adjustment disorders, anorexia nervosa, anxiety disorders, major depressive disorder, and other mental health disorders), infectious (6 conditions: infectious gastroenteritis, neutropenia, pneumonia, sepsis of newborn, septicemia, and urinary tract infections), and respiratory (2 conditions: asthma and bronchiolitis) (Figure 1). Most of the prevalent and costly medical conditions were newborn conditions (low birth weight, preterm newborn, and other perinatal conditions).

The variation in cost per encounter across hospitals was examined for 23 of the 25 most costly medical conditions that had an adequate number of hospitals and/or encounters (Table 3). Two mental health conditions (other mental health disorders [ICC, 0.28] and anxiety disorders [ICC, 0.19]) and 3 newborn conditions (intrauterine hypoxia and birth asphyxia [ICC, 0.27], other perinatal conditions [ICC, 0.17], and surfactant deficiency disorder [ie, respiratory distress syndrome in newborn; ICC, 0.17]) had the highest variation in cost across hospitals using the ICC method. The outlier hospital method, which was also used to evaluate variation in cost, revealed that more than 50% of the hospitals examined had a high proportion of high- or low-cost hospitalizations for 3 conditions (chemotherapy [25 of 30 hospitals], intrauterine hypoxia and birth asphyxia [14 of 24 hospitals], and other mental health disorders [18 of 30 hospitals]) (Table 3). The variation in cost per encounter across general hospitals was examined for 21 of the 25 most costly medical conditions (eTable 2 in the Supplement). The variation in cost (measured using ICC) for most conditions was similar to the variation found when examined across all hospitals. However, for some conditions, such as intrauterine hypoxia and birth asphyxia (ICC, 0.06) and other perinatal conditions (ICC, 0.06), the ICCs were substantially lower across general hospitals.

**Table 3.** Variation in Cost per Encounter Across Hospitals for the 25 Medical Conditions With the Highest Cumulative Cost Among Children in Ontario From 2014 to 2019

Condition	Hospitals included, No.^a^	ICC^b^	Outlier hospitals, No.^b^		
			Low cost	High cost	Total^c^
Newborn					
Intrauterine hypoxia and birth asphyxia	24	0.27	11	3	14
Other perinatal conditions	81	0.17	4	15	19
Surfactant deficiency disorder	39	0.17	13	4	17
Low birth weight	70	0.14	26	5	31
Preterm newborn	67	0.11	16	6	22
Transient tachypnea of newborn	65	0.11	17	6	23
Respiratory distress of newborn	58	0.11	10	9	19
Drug withdrawal syndrome in newborn	35	0.07	5	5	9
Neonatal hyperbilirubinemia	76	0.03	7	4	11
Extreme immaturity of newborn^d^	NA	NA	NA	NA	NA
Mental health					
Other mental health disorders	30	0.28	9	9	18
Anxiety disorders	29	0.19	6	2	8
Major depressive disorder	34	0.05	3	2	5
Adjustment disorders	28	0.05	2	3	5
Anorexia nervosa	11	0.03	2	0	2
Infectious					
Septicemia	22	0.10	5	3	8
Sepsis of newborn	33	0.07	3	6	9
Neutropenia	14	0.07	2	1	3
Pneumonia	52	0.06	8	4	12
Infectious gastroenteritis	46	0.03	5	2	5
Urinary tract infections	45	0.01	0	1	1
Respiratory					
Bronchiolitis	48	0.03	6	3	9
Asthma	46	0.02	0	1	1
Cancer					
Chemotherapy	30	0.12	22	11	25
Acute lymphoid leukemia^d^	NA	NA	NA	NA	NA

Abbreviations: ICC, intraclass correlation coefficient; NA, not applicable.

^a^ Number of hospitals in Ontario with >25 encounters for each corresponding condition during the study. Analysis of variation in cost was performed using encounter data from these hospitals only.

^b^ The ICCs and number of outlier hospitals were calculated using costs that were adjusted for patient age, sex, number of complex chronic conditions present, material deprivation, and rural vs urban classification.

^c^ Total number of distinct outlier hospitals for each condition. For some conditions, the total number does not equal the sum of low- and high-cost outlier hospitals because some of these hospitals were reported as both low- and high-cost outlier hospitals.

^d^ Analysis of variation in cost was not performed for extreme immaturity of newborn and acute lymphoid leukemia because there were <10 hospitals with >25 encounters for each of the conditions.

### Prevalence and Cost by Hospital Type

At general hospitals, the median annual volume of encounters was 53.6 (IQR, 4.0-757.2), whereas in pediatric hospitals, the median annual volume was 4117.1 (IQR, 1924.9-7704.4). The volume and cost of inpatient encounters among children in pediatric and general hospitals are shown in eTable 3 in the Supplement.

**Figure 2.**
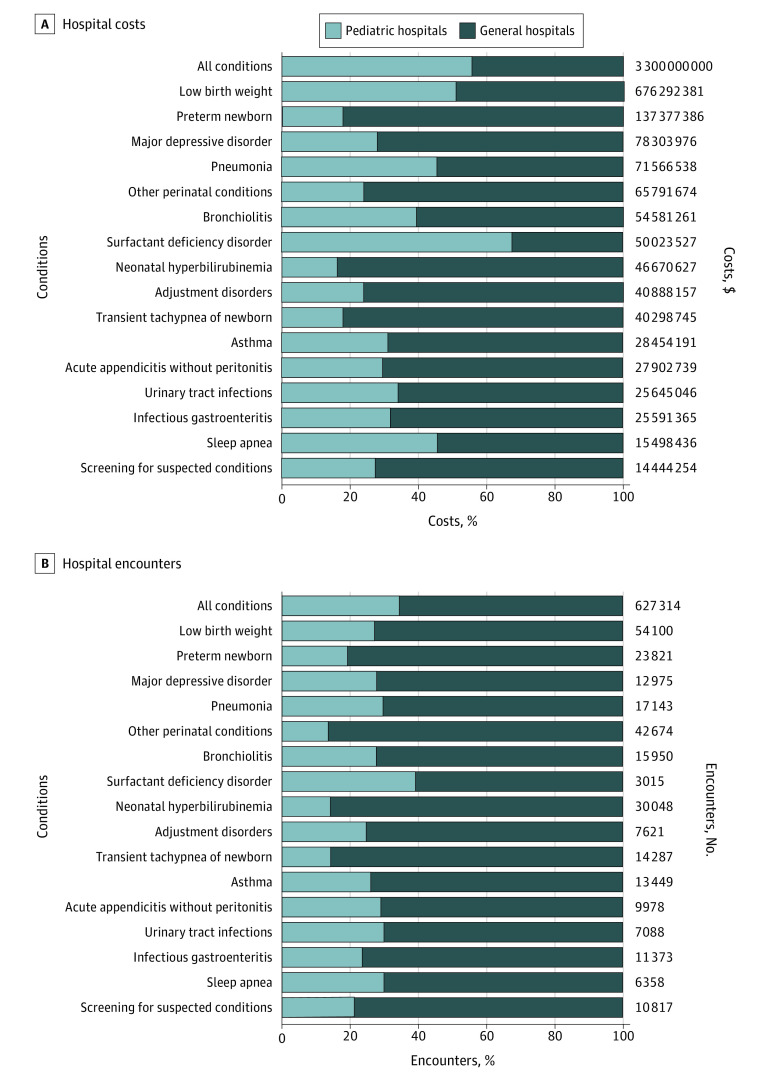
Costs and Encounters of the Most Costly and Prevalent Conditions in Pediatric and General Hospitals in Ontario, Canada, From 2014 to 2019 Percentage of hospital costs and encounters for all conditions and for the most costly and/or prevalent conditions (based on the top 25 most prevalent conditions and the top 25 most costly conditions identified for each hospital type in eTable 4 and eTable 5 in the Supplement). Costs were adjusted for inflation to 2018 US dollars (mean 2018 exchange rate: $0.77 US dollars = $1.00 Canadian dollar).

The rank order of the 25 most prevalent and 25 most costly conditions differed slightly between pediatric and general hospitals (eTable 4 and eTable 5 in the Supplement). In pediatric hospitals, the conditions with the highest prevalence and cost were low birth weight (68.2 per 1000 encounters; $348.1 million) and chemotherapy (28.4 per 1000 encounters; $34.2 million). In general hospitals, the most prevalent and costly conditions were low birth weight (95.9 per 1000 encounters) and other perinatal conditions (89.7 per 1000 encounters), and the most costly were low birth weight ($328.1 million) and preterm newborn ($112.3 million). The percentages of hospital costs and encounters that occurred in pediatric vs general hospitals for all conditions in total and for the most costly and/or most prevalent conditions identified in both hospital types are shown in Figure 2. Overall, inpatient encounters at general hospitals represented 65.0% of all hospital encounters and 43.8% of all hospital costs among children. With regard to the most costly and/or prevalent conditions in both hospital types, most of the hospital costs (59.2%) and encounters (77.0%) occurred in general hospitals.

## Discussion

This population-based cross-sectional study of health administrative data from Canada’s most populous province identified pediatric hospital conditions with high prevalence, cost, and/or variation in cost from 2014 to 2019. The study included 627 314 hospitalizations among children at all general and pediatric hospitals, finding that 65.0% of encounters and 43.8% of costs occurred at general hospitals. Several mental health and newborn conditions (low birth weight, preterm newborn, major depressive disorder, other perinatal conditions, and neonatal hyperbilirubinemia) had the highest prevalence, cost, and variation in cost across hospitals. Findings from this study can be combined with research priorities identified by patients, caregivers, and clinicians^38^ to develop a research agenda for investigators and funders conducting research involving hospitalized children. Furthermore, the findings may be used to prioritize hospital quality improvement efforts.

A separate study recently examined the prevalence and cost of conditions in US pediatric hospitals.^18^ Although it is difficult to compare the current study with the previous US study^18^ because of differences in health care systems,^39^ coding systems used (*ICD-10-CA* vs *ICD-10-CM*), and hospital types included, we observed several differences in the rank order of conditions. Newborn conditions such as low birth weight, which were ranked higher in cumulative cost in the current study, were ranked lower in cumulative cost in US pediatric hospitals,^18^ which likely reflects the large role of nonpediatric hospitals in neonatal care as well as potential *ICD-10* coding differences. The US study^18^ also identified fewer mental health conditions as prevalent and costly (2 mental health conditions within the top 50 list compared with 7 mental health conditions in the present study). In 1 study conducted in Ontario between 1991 to 1992 and 1997 to 1998,^20^ respiratory conditions, such as bronchiolitis and asthma, were the primary reasons for medical hospitalization among children younger than 15 years. In the current study, bronchiolitis was ranked sixth and asthma ranked eighth in the list of most prevalent conditions.

Several mental health conditions were identified among the 50 most prevalent and costly conditions, consistent with recent reports^40,41^ identifying mental disorders as one of the most common reasons for pediatric hospitalization. A recent study^41^ reported a 60% increase in hospitalizations for mental disorders among children and youths in Canada from 2008 to 2009 and 2018 to 2019. Beyond the health care system burden, 2 mental health conditions (anxiety disorders and other mental health disorders) also had high variation in cost across hospitals, with the broad category of other mental health disorders having the highest ICC (0.28) of all costly medical conditions (specific mental health diagnoses are available in eTable 1 in the Supplement). The high cost and prevalence of mental health conditions in pediatric hospitalization may be owing to a lack of effective interventions and the presence of barriers to accessing mental health services, including long waiting times for counseling and therapy, lack of available mental health professionals, and challenges regarding access to community-based mental health services.^42,43,44,45^ In Ontario, rates of outpatient visits have modestly increased, whereas emergency department visits and hospitalizations for mental health among children have substantially increased between 2009 and 2017.^46^ This difference suggests the presence of barriers to outpatient mental health care and an increase in the prevalence^43^ and burden of mental illnesses that are severe enough to require hospitalization.^46^ Our study findings highlight the need to improve care standardization across hospitals and evaluate new mental health interventions for children to identify those that are most cost-effective.

Newborn conditions, such as low birth weight and preterm newborn, were also the most prevalent and costly conditions among hospitalized children. We found that almost one-half (44.8%) of the inpatient encounters occurred among children younger than 30 days. Similar findings were observed in previous Canadian studies.^20,40,47^ Over the last few decades, research in the field of neonatology has produced advancements in neonatal care, which has been associated with improvements in survival and outcomes among infants.^48,49^ The high cost and prevalence of newborn conditions observed in our study suggests the need for research to identify cost-effective interventions for this population. In addition to high cost and prevalence, several newborn conditions (eg, intrauterine hypoxia and birth asphyxia) had high variation in cost across hospitals. These conditions represent areas in which better understanding of clinical practice variation is needed to identify whether future research or quality improvement initiatives are required to address them.

Most pediatric hospitalizations in Ontario (65.0%) occurred in general hospitals, which is similar to the 70% previously reported in the US.^17^ The high prevalence and cost of encounters observed in general hospitals suggest the need for future research to focus on ways to better manage these conditions among children admitted to general hospitals and to prioritize quality improvement efforts toward these areas. Most published studies on hospital use or ways to improve hospital care among children have only included pediatric hospitals.^18,30,50,51,52^ The lack of inclusion of general hospitals (including community hospitals) in research leaves important clinical care questions in these hospitals unanswered.^53^ Furthermore, because of differences in hospital characteristics (eg, practice settings), quality improvement initiatives based on research conducted in pediatric hospitals may not be applicable to general hospitals.^53^ It is important that general hospitals be included in research involving hospitalized children to ensure findings are generalizable and reflect the hospital setting in which most children receive care.^53^

### Limitations

This study has several limitations. First, errors may have occurred when coding the discharge diagnosis, which may have resulted in misclassification of conditions. Second, hospitalization costs were estimated using provincial mean unit costs, and the amount of resources consumed was estimated using the patient’s resource intensity weight. Therefore, the costs do not reflect the actual costs of the resources used and may underestimate the extent of the cost variation.^26,54^ Third, unmeasured factors (eg, disease severity) may have accounted for a portion of the variation in cost across hospitals. Fourth, a few nonspecific conditions, such as other mental health disorders, had high prevalence, cost, and/or variation in cost across hospitals. Future condition-specific research could focus on examining the specific diagnostic codes underlying these nonspecific conditions. Fifth, our system-level analysis did not examine the condition-specific sources of cost or variation in cost (eg, length of stay). Future condition-specific studies could examine these sources. Sixth, the data used in this study were reported before the COVID-19 pandemic.

## Conclusions

This population-based cross-sectional study used health administrative data from Ontario, Canada, to identify conditions with high prevalence, cost, and/or variation in cost as priorities for future research. Several newborn and mental health conditions (low birth weight, preterm newborn, major depressive disorder, other perinatal conditions, and neonatal hyperbilirubinemia) were identified as having the highest prevalence, cost, and variation in cost across hospitals. Findings from this study can be used by investigators and funders to develop a research agenda that includes general hospitals, with the goal of improving the evidence base and outcomes for hospitalized children. Furthermore, the findings may help prioritize hospital quality improvement initiatives to ensure high-quality hospital care is provided to children.

## Notes

Supplement. **eFigure.** Flow Diagram of Cohort Construction **eTable 1.** Top 10 *ICD-10-CA* Most Responsible Diagnosis Codes That Led to Hospital Encounters for Each Nonspecific Pediatric Condition Identified in Children With Inpatient Encounters (From Table 2) in Ontario, 2014-2019 **eTable 2.** Variation in Cost per Encounter Across General Hospitals for the 25 Conditions With the Highest Cumulative Cost Among Children in Ontario, 2014-2019 **eTable 3.** Volume and Cost of Inpatient Encounters in Pediatric Hospitals and General Hospitals, 2014-2019 **eTable 4.** Prevalence and Cost of the 25 Most Costly and 25 Most Prevalent Conditions Among Children With Inpatient Encounters in Pediatric Hospitals in Ontario, 2014-2019 **eTable 5.** Prevalence and Cost for the 25 Most Costly and 25 Most Prevalent Conditions Among Children With Inpatient Encounters in General Hospitals in Ontario, 2014-2019
