# Supplementary material for: Prevalence, Cost, and Variation in Cost of Pediatric Hospitalizations in Ontario, Canada
Source: JAMA Netw Open. 2022 Feb 9;5(2):e2147447. doi: 10.1001/jamanetworkopen.2021.47447 (PMC8829658; doi:10.1001/jamanetworkopen.2021.47447)
Supplement: Supplement. — eFigure. Flow Diagram of Cohort Construction eTable 1. Top 10 ICD-10-CA Most Responsible Diagnosis Codes That Led to Hospital Encounters for Each Nonspecific Pediatric Condition Identified in Children With Inpatient Encounters (From Table 2) in Ontario, 2014-2019 eTable 2. Variation in Cost per Encounter Across General Hospitals for the 25 Conditions With the Highest Cumulative Cost Among Children in Ontario, 2014-2019 eTable 3. Volume and Cost of Inpatient Encounters in Pediatric Hospitals and General Hospitals, 2014-2019 eTable 4. Prevalence and Cost of the 25 Most Costly and 25 Most Prevalent Conditions Among Children With Inpatient Encounters in Pediatric Hospitals in Ontario, 2014-2019 eTable 5. Prevalence and Cost for the 25 Most Costly and 25 Most Prevalent Conditions Among Children With Inpatient Encounters in General Hospitals in Ontario, 2014-2019 [file jamanetwopen-e2147447-s001.pdf]

Supplementary Online Content

Gill PJ, Thavam T, Anwar MR, et al. Prevalence, cost, and variation in cost of pediatric hospitalizations in Ontario, Canada. *JAMA Netw Open*. 2022;5(2):e2147447. doi:10.1001/jamanetworkopen.2021.47447

eFigure. Flow Diagram of Cohort Construction

eTable 1. Top 10 *ICD-10-CA* Most Responsible Diagnosis Codes That Led to Hospital Encounters for Each Nonspecific Pediatric Condition Identified in Children With Inpatient Encounters (From Table 2) in Ontario, 2014-2019

eTable 2. Variation in Cost per Encounter Across General Hospitals for the 25 Conditions With the Highest Cumulative Cost Among Children in Ontario, 2014-2019

eTable 3. Volume and Cost of Inpatient Encounters in Pediatric Hospitals and General Hospitals, 2014-2019

eTable 4. Prevalence and Cost of the 25 Most Costly and 25 Most Prevalent Conditions Among Children With Inpatient Encounters in Pediatric Hospitals in Ontario, 2014-2019

eTable 5. Prevalence and Cost for the 25 Most Costly and 25 Most Prevalent Conditions Among Children With Inpatient Encounters in General Hospitals in Ontario, 2014-2019

This supplementary material has been provided by the authors to give readers additional information about their work.

© 2022 Gill PJ et al. *JAMA Network Open*.

eFigure 1. Flow Diagram of Cohort Construction

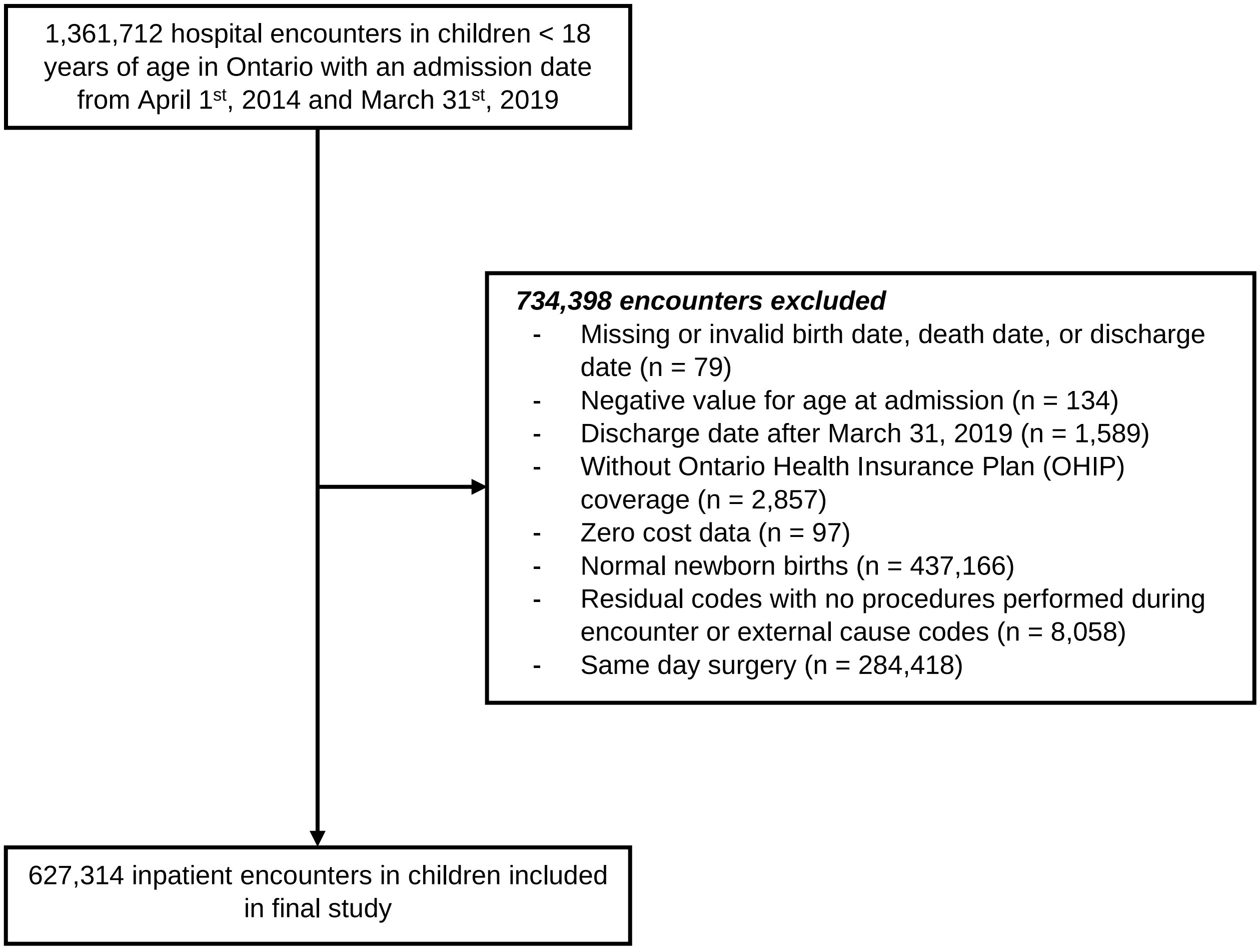

© 2022 Gill PJ et al. *JAMA Network Open*.

eTable 1. Top 10 *ICD-10-CA* Most Responsible Diagnosis Codes That Led to Hospital Encounters for Each Nonspecific Pediatric Condition Identified in Children With Inpatient Encounters (From Table 2) in Ontario, 2014-2019

**Condition**	**Total Number of Encounters**	**ICD-10-CA Diagnosis Code Descriptions[a]**	**No. (%) of Encounters[b]**
Other perinatal conditions	42,674	Other heavy for gestational age infants	13,627 (31.9)
		Post-term infant, not heavy for gestational age	10,670 (25.0)
		Exceptionally large baby	3,660 (8.6)
		Other cardiovascular disorders originating in the perinatal period	3,580 (8.4)
		Condition originating in the perinatal period, unspecified	870 (2.0)
		Fetus and newborn affected by other malpresentation, malposition and disproportion during labour and delivery	721 (1.7)
		Fetus and newborn affected by other compression of umbilical cord	705 (1.7)
		Apparent life threatening event of infant	628 (1.5)
		Fetus and newborn affected by maternal use of drugs of addiction	589 (1.4)
		Fetus and newborn affected by breech delivery and extraction	520 (1.2)
		Other ICD-10-CA diagnosis codes	7,104 (16.6)
Other congenital anomalies	3,077	Other specified congenital malformations of skin	259 (8.4)
		Congenital malformation syndromes predominantly affecting facial appearance	163 (5.3)
		Unstable hip	151 (4.9)
		Preauricular sinus and cyst	136 (4.4)
		Other specified congenital malformations	110 (3.6)
		Other congenital malformations of lower limb(s), including pelvic girdle	101 (3.3)
		Other reduction defects of lower limb(s)	86 (2.8)
		Other congenital malformations of upper limb(s), including shoulder girdle	76 (2.5)
		Congenital deformity of sternocleidomastoid muscle	67 (2.2)
		Other specified congenital musculoskeletal deformities	66 (2.1)
		Other ICD-10-CA diagnosis codes	1,862 (60.5)
Other mental health disorders	3,168	Other symptoms and signs involving emotional state	2,073 (65.4)

© 2022 Gill PJ et al. *JAMA Network Open*.

Condition	Total Number of Encounters	ICD-10-CA Diagnosis Code Descriptions[a]	No. (%) of Encounters[b]
		Other eating disorders	309 (9.8)
		Eating disorder, unspecified	284 (9.0)
		Dissociative [conversion] disorder, unspecified	199 (6.3)
		Gender identity disorder, unspecified	35 (1.1)
		Somatoform disorder, unspecified	31 (1.0)
		Somatization disorder	29 (0.9)
		Other gender identity disorders	24 (0.8)
		Mental disorder, not otherwise specified	16 (0.5)
		Other somatoform disorders	16 (0.5)
		Other ICD-10-CA diagnosis codes	152 (4.8)
Screening for suspected conditions	10,817	Observation for other suspected diseases and conditions	9,981 (92.3)
		Other specified abnormal findings of blood chemistry	172 (1.6)
		Observation for suspected disease or condition, unspecified	108 (1.0)
		Observation for other suspected cardiovascular diseases	106 (1.0)
		Observation for suspected toxic effect from ingested substance	105 (1.0)
		Abnormal results of kidney function studies	83 (0.8)
		Observation for suspected nervous system disorder	68 (0.6)
		Abnormal results of liver function studies	38 (0.4)
		Observation for suspected mental and behavioural disorders	33 (0.3)
		Special screening examination for other specified diseases and disorders	15 (0.1)
		Other ICD-10-CA diagnosis codes	108 (1.0)
Other lower respiratory disease	3,533	Other specified respiratory disorders	1,626 (46.0)
		Unspecified acute lower respiratory infection	829 (23.5)
		Other and unspecified abnormalities of breathing	767 (21.7)
		Other disorders of lung	77 (2.2)
		Haemoptysis	72 (2.0)
		Pulmonary oedema	26 (0.7)
		Respiratory disorder, unspecified	24 (0.7)

© 2022 Gill PJ et al. *JAMA Network Open*.

Condition	Total Number of Encounters	ICD-10-CA Diagnosis Code Descriptions[a]	No. (%) of Encounters[b]
		Interstitial pulmonary disease, unspecified	21 (0.6)
		Haemorrhage from other sites in respiratory passages	20 (0.6)
		Periodic breathing	17 (0.5)
		Other ICD-10-CA diagnosis codes	54 (1.5)
Other aftercare	4,738	Convalescence following surgery	3,244 (68.5)
		Convalescence following other treatment	330 (7.0)
		Other specified medical care NEC	236 (5.0)
		Preparatory care for subsequent treatment, not elsewhere classified	181 (3.8)
		Palliative care	132 (2.8)
		Convalescence following treatment of fracture	98 (2.1)
		Follow-up examination after surgery for other conditions	76 (1.6)
		Blood transfusion (without reported diagnosis)	63 (1.3)
		Pain management planning	58 (1.2)
		Other specified surgical follow-up care	53 (1.1)
		Other ICD-10-CA diagnosis codes	267 (5.6)
Other convulsions	2,954	Other and unspecified convulsions	2,138 (72.4)
		Seizure disorder, so described	816 (27.6)
Respiratory problems after birth, other	2,882	Other specified respiratory conditions of newborn	2,315 (80.3)
		Respiratory condition of newborn, unspecified	567 (19.7)

Abbreviations: ICD-10-CA, International Statistical Classification of Diseases and Related Health Problems, Tenth Revision Canada; NEC, not elsewhere classified.
[a] Indicates the descriptions for the top 10 ICD-10-CA diagnosis codes that led to hospital encounters for each non-specific pediatric condition. The remainder ICD-10-CA codes that led to encounters for the corresponding condition was grouped under *'Other ICD-10-CA diagnosis codes'* for that condition.
[b] The data reflects the number and percentage of encounters for each ICD-10-CA diagnosis code within the corresponding condition.

© 2022 Gill PJ et al. *JAMA Network Open*.

eTable 2. Variation in Cost per Encounter Across General Hospitals for the 25 Conditions With the Highest Cumulative Cost Among Children in Ontario, 2014-2019

Condition	Number of Hospitals Included[a]	ICC[b]	Number of Outlier Hospitals[b]		
			Low Cost	High Cost	Total[c]
**Newborn condition**					
Intrauterine hypoxia and birth asphyxia	17	0.06	1	3	4
Other perinatal conditions	73	0.06	2	12	14
Surfactant deficiency disorder	31	0.12	5	4	9
Low birth weight	62	0.13	20	4	24
Preterm newborn	60	0.10	17	4	21
Transient tachypnea of newborn	57	0.09	16	4	20
Respiratory distress of newborn	51	0.07	9	9	18
Drug withdrawal syndrome in newborn	30	0.03	2	5	6
Neonatal hyperbilirubinemia	68	0.02	5	3	8
Extreme immaturity of newborn[d]	-	-	-	-	-
**Mental health condition**					
Other mental health disorders	25	0.33	7	8	15
Anxiety disorders	24	0.22	6	1	7
Major depressive disorder	29	0.05	3	3	6
Adjustment disorders	23	0.06	2	5	7
Anorexia nervosa[d]	-	-	-	-	-
**Infectious condition**					
Septicemia	18	0.00	1	2	3
Sepsis of newborn	27	0.03	2	2	4
Neutropenia[d]	-	-	-	-	-
Pneumonia	47	0.03	2	5	7
Infectious Gastroenteritis	41	0.00	1	0	1
Urinary tract infections	40	0.00	0	0	0
**Respiratory condition**					
Bronchiolitis	43	0.01	1	2	3
Asthma	41	0.02	0	3	3
**Cancer**					
Chemotherapy	23	0.13	5	10	15
Acute lymphoid leukemia[d]	-	-	-	-	-

Abbreviations: ICC, Intraclass correlation coefficient.
[a] For each condition, it indicates the number of general hospitals in Ontario with > 25 encounters for the corresponding condition during the study. Variation in cost analysis was performed using encounter data from these hospitals only.
[b] The ICCs and number of outlier hospitals were calculated using costs that were adjusted for patient's age, sex, number of complex chronic conditions present, material deprivation, and rural-urban classification.
[c] Indicates the total number of unique outlier general hospitals for each condition. For some conditions, the total number will not equal to the sum of low cost and high cost outlier general hospitals, as some of these hospitals were reported in both low and high cost outlier hospital.
[d] Variation in cost analysis was not performed for extreme immaturity of newborn, anorexia nervosa, neutropenia, and acute lymphoid leukemia, because there were less than 10 general hospitals with > 25 encounters for each of the condition to conduct this analysis.

© 2022 Gill PJ et al. *JAMA Network Open*.

eTable 3. Volume and Cost of Inpatient Encounters in Pediatric Hospitals and General Hospitals, 2014-2019

	**Pediatric Hospitals**	**General Hospitals**
**Number of encounters, No.**	219,311	408,003
**Percent of encounters from all encounters in children, %**	35.0	65.0
**Cost of encounters, $USD[a]**	1,851,916,589	1,443,735,382
**Percent of cost of encounters from the total cost of encounters in children, %**	56.2	43.8
**Annual volume of encounters, median (IQR)**	4,117.1 (1,924.9, 7,704.4)	53.6 (4.0, 757.2)

Abbreviations: USD, US dollars; IQR, interquartile range.
[a] Costs adjusted for inflation to 2018 US dollars (mean 2018 exchange rate: $0.77 US dollars = $1.00 Canadian dollar).

© 2022 Gill PJ et al. *JAMA Network Open*.

eTable 4. Prevalence and Cost of the 25 Most Costly and 25 Most Prevalent Conditions Among Children With Inpatient Encounters in Pediatric Hospitals in Ontario, 2014-2019

Condition	Type	Rank Based on		Number of Encounters		Cost ($ USD)[d]	
		Total Cost	Number of Encounters[a]	Total[b]	Prevalence[c]	Total	Per Encounter Median (IQR)
Low birth weight	Medical	1	1	14,962	68.2	348,173,239	6,435 (1,928-20,595)
Chemotherapy	Medical	2	2	6,222	28.4	34,211,554	4,384 (3,754-4,544)
Surfactant deficiency disorder	Medical	3	38	1,195	5.4	33,819,387	11,875 (6,202-27,321)
Pneumonia	Medical	4	4	5,175	23.6	32,831,102	3,838 (2,781-5,562)
Preterm newborn	Medical	5	5	4,737	21.6	25,080,526	1,312 (1,252-5,897)
Acute lymphoid leukemia	Medical	6	61	835	3.8	23,196,530	19,114 (13,943-30,242)
Other congenital anomalies	Surgical	7	26	1,889	8.6	22,835,579	3,750 (2,377-6,232)
Major depressive disorder	Medical	8	8	3,669	16.7	22,155,173	5,779 (5,341-6,735)
Anorexia nervosa	Medical	9	37	1,227	5.6	22,108,487	18,399 (16,329-18,752)
Bronchiolitis	Medical	10	6	4,497	20.5	21,750,457	2,626 (2,491-3,972)
Necrotizing enterocolitis	Medical/Surgical	11	148	324	1.5	21,515,167	24,992 (14,961-78,015)
Scoliosis	Surgical	12	45	1,020	4.7	20,118,088	18,884 (12,943-19,834)
Complications of surgical procedures or medical care	Medical/Surgical	13	14	2,575	11.7	18,346,151	2,686 (1,802-5,790)
Respiratory failure	Medical	14	98	527	2.4	17,777,414	18,452 (7,403-45,473)
Tetralogy of fallot	Medical/Surgical	15	97	536	2.4	17,518,377	18,242 (4,607-30,921)
Transposition of great vessels	Medical/Surgical	16	112	444	2.0	16,377,101	21,649 (3,636-45,820)
Other perinatal conditions	Medical	17	3	6,066	27.7	16,088,503	1,065 (1,030-1,326)
Gastroschisis & Exomphalos	Surgical	18	156	300	1.4	15,594,173	24,370 (1,332-68,466)
Congenital tracheoesophageal disorders	Surgical	19	190	215	1.0	15,406,927	26,902 (3,786-67,707)
Hypoplastic left heart syndrome	Medical/Surgical	20	182	234	1.1	15,256,568	15,340 (1,758-54,583)

© 2022 Gill PJ et al. *JAMA Network Open*.

Condition	Type	Rank Based on		Number of Encounters		Cost ($ USD)[d]	
		Total Cost	Number of Encounters[a]	Total[b]	Prevalence[c]	Total	Per Encounter Median (IQR)
Intrauterine hypoxia and birth asphyxia	Medical	21	46	989	4.5	15,250,690	10,189 (4,070-16,635)
Neutropenia	Medical	22	22	2,055	9.4	14,438,459	4,292 (3,737-7,389)
Septicemia	Medical	23	60	840	3.8	13,376,766	7,948 (4,615-15,634)
Cystic fibrosis	Medical	24	71	755	3.4	13,143,030	14,365 (12,436-16,173)
Extreme immaturity of newborn	Medical	25	231	159	0.7	12,852,505	63,389 (28,916-101,987)
Fracture of lower limb	Surgical	28	17	2,328	10.6	11,886,094	3,953 (3,334-6,744)
Seizures with and without intractable epilepsy	Medical	29	12	2,747	12.5	11,786,414	1,905 (1,827-3,184)
Acute appendicitis with peritonitis	Surgical	30	23	2,033	9.3	11,115,823	4,045 (3,453-5,676)
Adjustment disorders	Medical	36	25	1,926	8.8	10,203,070	5,067 (4,433-6,314)
Asthma	Medical	39	9	3,565	16.3	8,971,143	1,739 (1,698-2,448)
Urinary tract infections	Medical	41	19	2,142	9.8	8,854,935	3,170 (2,844-3,916)
Acute appendicitis without peritonitis	Surgical	44	10	2,949	13.4	8,352,567	2,655 (2,519-2,832)
Infectious Gastroenteritis	Medical	46	13	2,723	12.4	8,286,627	1,862 (1,721-2,896)
Supracondylar fracture of humerus	Surgical	49	11	2,861	13.0	8,201,937	2,943 (2,829-2,973)
Acute upper respiratory infection	Medical	51	21	2,088	9.5	7,806,877	2,092 (1,971-2,582)
Neonatal hyperbilirubinemia	Medical	52	7	4,449	20.3	7,798,536	1,447 (1,064-1,514)
Transient tachypnea of newborn	Medical	55	20	2,122	9.7	7,364,327	1,946 (1,522-3,949)
Sleep apnea	Surgical	57	24	1,932	8.8	7,116,573	1,842 (1,828-1,928)
Other aftercare	Medical/Surgical	63	15	2,398	10.9	6,840,110	1,275 (1,186-2,759)
Fracture of upper limb	Surgical	64	18	2,296	10.5	6,582,377	2,930 (2,281-3,034)
Screening for suspected conditions	Medical	100	16	2,346	10.7	4,031,916	1,233 (1,036-1,378)

Abbreviations: USD, US dollars; IQR, interquartile range.
[a] Also known as prevalence rank in this study.

© 2022 Gill PJ et al. *JAMA Network Open*.

[b] Total number of encounters for the condition over the study period.
[c] Condition-specific prevalence calculated per 1000 encounters.
[d] Costs adjusted for inflation to 2018 US dollars (mean 2018 exchange rate: $0.77 US dollars = $1.00 Canadian dollar).

© 2022 Gill PJ et al. *JAMA Network Open*.

eTable 5. Prevalence and Cost for the 25 Most Costly and 25 Most Prevalent Conditions Among Children With Inpatient Encounters in General Hospitals in Ontario, 2014-2019

Condition	Type	Rank Based on		Number of Encounters		Cost ($ USD)[d]	
		Total Cost	Number of Encounters[a]	Total[b]	Prevalence[c]	Total	Per Encounter Median (IQR)
Low birth weight	Medical	1	1	39,138	95.9	328,119,142	2,243 (1,227-11,834)
Preterm newborn	Medical	2	4	19,084	46.8	112,296,860	2,706 (1,255-6,140)
Major depressive disorder	Medical	3	9	9,306	22.8	56,148,803	5,849 (5,306-6,735)
Other perinatal conditions	Medical	4	2	36,608	89.7	49,703,171	1,036 (1,026-1,236)
Neonatal hyperbilirubinemia	Medical	5	3	25,599	62.7	38,872,091	1,447 (1,064-1,500)
Pneumonia	Medical	6	6	11,968	29.3	38,735,436	2,870 (2,696-3,168)
Drug withdrawal syndrome in newborn	Medical	7	27	2,939	7.2	33,321,467	9,473 (8,793-13,395)
Transient tachypnea of newborn	Medical	8	5	12,165	29.8	32,934,418	1,541 (1,428-3,186)
Bronchiolitis	Medical	9	7	11,453	28.1	32,830,804	2,602 (2,375-2,700)
Adjustment disorders	Medical	10	17	5,695	14.0	30,685,087	5,113 (4,675-6,314)
Anxiety disorders	Medical	11	24	3,285	8.1	20,050,617	6,448 (5,394-7,030)
Acute appendicitis without peritonitis	Surgical	12	13	7,029	17.2	19,550,172	2,655 (2,519-2,832)
Asthma	Medical	13	8	9,884	24.2	19,483,048	1,723 (1,623-1,982)
Infectious Gastroenteritis	Medical	14	10	8,650	21.2	17,304,738	1,734 (1,706-1,889)
Urinary tract infections	Medical	15	20	4,946	12.1	16,790,111	3,112 (2,830-3,465)
Respiratory distress of newborn	Medical	16	15	6,339	15.5	16,660,328	1,541 (1,428-2,651)
Surfactant deficiency disorder	Medical	17	44	1,820	4.5	16,204,139	4,050 (1,561-10,120)
Feeding difficulties	Medical/Surgical	18	19	5,384	13.2	15,547,809	1,354 (1,030-2,264)
Newborn respiratory failure	Medical	19	18	5,664	13.9	13,754,596	1,269 (1,123-2,335)
Hypertrophy of tonsils and adenoids	Surgical	20	12	7,070	17.3	13,456,130	1,842 (1,828-1,907)
Neonatal hypoglycemia	Medical	21	14	6,784	16.6	13,110,005	1,078 (1,030-2,134)

© 2022 Gill PJ et al. *JAMA Network Open*.

Condition	Type	Rank Based on		Number of Encounters		Cost ($ USD)[d]	
		Total Cost	Number of Encounters[a]	Total[b]	Prevalence[c]	Total	Per Encounter Median (IQR)
Sepsis of newborn	Medical	22	38	2,257	5.5	12,850,840	4,635 (2,933-6,330)
Schizophrenia and psychotic disorders	Medical	23	68	1,023	2.5	11,347,210	10,769 (9,374-11,384)
Acute appendicitis with peritonitis	Surgical	24	31	2,367	5.8	11,055,001	4,007 (3,923-4,443)
Viral infection	Medical	25	21	4,844	11.9	10,510,770	1,898 (1,826-2,063)
Screening for suspected conditions	Medical	26	11	8,471	20.8	10,412,338	1,036 (1,026-1,218)
Infant of diabetic mother	Medical	29	16	5,884	14.4	9,674,910	1,065 (1,030-1,236)
Sleep apnea	Surgical	31	22	4,426	10.8	8,381,863	1,842 (1,828-1,907)
ABO Hemolytic disease	Medical	37	25	3,267	8.0	6,982,068	1,858 (1,682-2,190)
Tongue tie	Surgical	52	23	4,354	10.7	4,980,370	1,036 (1,026-1,078)

Abbreviations: USD, US dollars; IQR, interquartile range; ABO, ABO blood group system.
[a] Also known as prevalence rank in this study.
[b] Total number of encounters for the condition over the study period.
[c] Condition-specific prevalence calculated per 1000 encounters.
[d] Costs adjusted for inflation to 2018 US dollars (mean 2018 exchange rate: $0.77 US dollars = $1.00 Canadian dollar).

© 2022 Gill PJ et al. *JAMA Network Open*.
